# Antimicrobial photocatalysis using bio-hydrothermally synthesized Zinc oxide nanoparticles in the management of periodontitis: a prospective split-mouth, double-blind, randomized, controlled clinical trial*

**DOI:** 10.1590/1678-7757-2023-0271

**Published:** 2023-12-18

**Authors:** C. Afigith MATHEW, H. R. VEENA, P. SHUBHA, Riya Achamma DANIEL

**Affiliations:** 1 K. L. E. Society’s Institute of Dental Sciences Department of Periodontics Bangalore Karnataka India K. L. E. Society’s Institute of Dental Sciences, Department of Periodontics, Bangalore, Karnataka 560022, India.; 2 Mangalore University Department of Material Science Mangalore Karnataka India Mangalore University, Department of Material Science, Mangalagangotri, Mangalore, Karnataka 574199, India.; 3 MIOT International Department of Dental Surgery Chennai Tamil Nadu India MIOT International, Department of Dental Surgery, Chennai, Tamil Nadu 600089, India.

**Keywords:** Periodontitis, Photocatalysis, Visible light, Zinc Oxide, Nanoparticles

## Abstract

The antimicrobial activity of metallic nanoparticles (NPs) has been confirmed to fight a broad spectrum of microorganisms, through antimicrobial effects that are amplified when these particles are irradiated with light of the proper wavelength. This is the first study to use phytoconjugated Zinc oxide (ZnO) NPs containing traces of active biomolecules derived from *Emblica officinalis* (*E. officinalis*) plant extract in antimicrobial photocatalysis (PCT) during non-surgical periodontal therapy.

**Objectives:**

This study aimed to evaluate the effects of repeated PCT application in the treatment of periodontitis, using a gel containing bio-hydrothermally synthesized ZnO NPs and visible light as an adjunct to scaling and root planing (SRP).

**Methodology:**

In total, 16 systemically healthy volunteers with stage 3 grade B generalized periodontitis were recruited for this prospective double blind, randomized placebo-controlled trial. After receiving SRP, the subjects received the following interventions in a split-mouth design at baseline, 1 week and 1 month: Group 1 – Placebo gel + Sham PCT; Group 2 – Nano ZnO gel + Sham PCT; Group 3 – Placebo gel + PCT; and Group 4 – Nano ZnO gel + PCT. The site-specific profile of *Porphyromonas gingivalis* in the subgingival plaque and clinical parameters (Plaque Index, Gingival Index, Gingival Bleeding Index, Probing pocket Depth and Clinical Attachment Level) were assessed at baseline, 1 month and 3 months.

**Results:**

All interventions tested caused participants’ clinical and microbiological parameters to generally improve after 3 months. Subjects who received the Nano ZnO gel + PCT combination showed a sustained and progressive improvement in their treatment outcomes, a result that presented statistically significant differences from the outcomes obtained through the remaining interventions, at all time points during the study period.

**Conclusions:**

The repeated application of PCT using bio-hydrothermally synthesized ZnO NPs can effectively complement SRP in the non-surgical treatment of Periodontitis.

## Introduction

The main objective of periodontal therapy is to prevent periodontitis by suppressing or eliminating periodontopathogenic bacteria. Even though scaling and root planing (SRP) and anti-infective chemotherapeutics are the conventional instruments used to treat periodontitis, they have their own drawbacks, such as causing systemic adverse effects and the development of bacterial resistance.^1^Given this situation, better therapeutic adjuncts are needed to improve the treatment outcomes of periodontitis.

Antimicrobial photocatalysis (PCT) is a process in which semiconductor nanoparticles (NPs) are irradiated with a light source, generating reactive oxygen species (ROS) in order to kill various types of microorganisms.^2^The antimicrobial activity of metal NPs has been confirmed to fight a wide range of broad spectrum microorganisms, through antimicrobial effects that are amplified when these particles are irradiated with light of the proper wavelength. This process is a viable alternative to antimicrobial photodynamic therapy (PDT) in the treatment of periodontal diseases.^3^This is the first study to use photocatalysts in periodontal therapy, as these materials are more commonly used as antifungal, antimicrobial or anticancer agents in the medical, environmental and energy fields, including in self-cleaning surfaces, air and water purification systems and sterilization processes.

**Figure 1:**
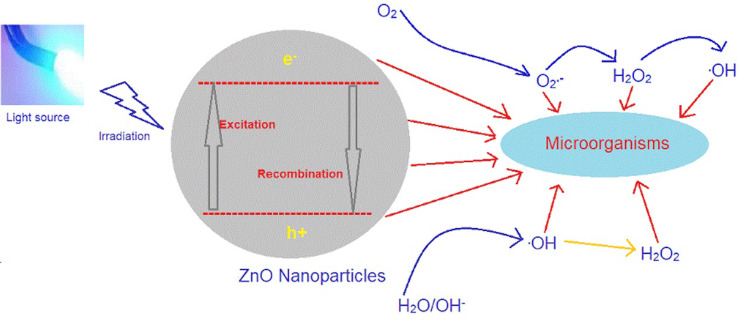
Mechanism of Antimicrobial Photocatalysis using ZnO NPs

Zinc oxide (ZnO), an antibacterial metal oxide, is widely used in Dentistry for indirect pulp capping and periodontal dressings, and as a temporary filling material or root canal sealer. ZnO is an n-type semiconductor metal oxide with a wide band-gap of 3.37ev and is considered a GRAS (Generally Regarded as Safe) substance by the US-FDA.^4^Recent evidence suggests that some of the attributes of ZnO NPs, such as the promising arrangement of its electronic structure, light absorption properties, and charge transport characteristics, make it possible to use it as a photosensitizer. ZnO NPs get photocatalyzed under both ultra-violet and visible light irradiation, releasing ROS, which eventually causes bacterial cell death (Figure 1).^4^

The demand for natural biomaterials has recently grown, since these substances are biodegradable, biocompatible, readily available and less toxic. In recent years, nanotechnology has become a new strategy to prevent the re-emergence of infectious diseases and the development of antibiotic-resistant strains, especially Gram-negative microorganisms. ZnO NPs with tailor-made properties for biomedical applications, which are in high demand, are synthesized using different methods, including physical, chemical and biological ones.^5^

In the bio-hydrothermal synthesis process, plant-derived active biomolecules are used as reducing and capping agents within the hydrothermal reaction system, which yields highly bioactive and biocompatible NPs with any required morphology and characteristics.^6^

Considering this phenomenon to be an advantage, this study aimed to evaluate the microbiological and clinical effects of PCT with bio-hydrothermally synthesized ZnO NPs and visible light when used as an adjunct to SRP in the non-surgical treatment of periodontitis.

## Methodology

The sample size was estimated using the G*Power 3.0.10 software, setting the alpha level at 0.05 and power at 80%. The effective sample size was calculated as 0.20. Based on these criteria, the ideal number of participants in each group was calculated to be 16, which generated a total sample size of 64.

**Figure 2:**
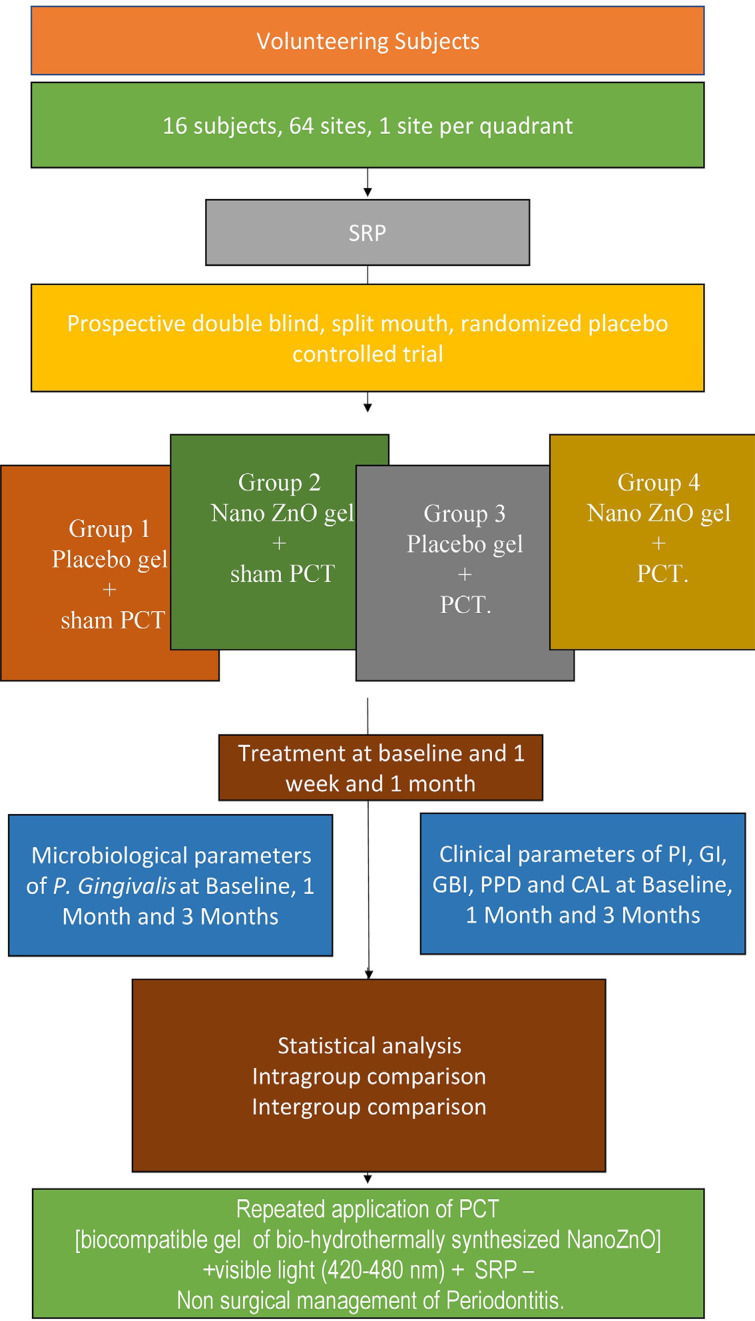
Study Flow Diagram

Following clearance from the Institutional Ethics Committee (Certificate No: KIDS/IEC/NOV-2019/36), 16 volunteering subjects who met the inclusion and exclusion criteria were recruited for this prospective double-blind, split-mouth, randomized placebo controlled trial after signing an informed consent document, in accordance with the Declaration of Helsinki (2013) (Figure 2) (Clinical Trials Registry of India Registration No: CTRI/2019/12/022458).

A single examiner enrolled systemically healthy individuals aged 30-50 years, diagnosed with stage 3 grade B generalized periodontitis, with a minimum of 20 teeth and at least one site with a probing pocket depth (PPD) of >5mm and clinical attachment level (CAL) of 2mm or greater in each of the four quadrants.^7^ This study did not include subjects who had undergone periodontal therapy or were administered antibiotics or immunosuppressants until 6 months before the study began, chronic smokers, alcoholics, non-smoking tobacco users, subjects with acute illnesses/acute intraoral lesions, pregnant women and lactating mothers, and medically compromised subjects.

After a clinical examination the recording of clinical and microbiological parameters at baseline and a full mouth SRP were conducted and each subject’s 4 quadrants were randomly assigned to one of the following treatment groups through a simple, computer-generated randomization technique:

Group 1 (control) – Application of placebo gel followed by sham PCT (directing the light cure device without turning on the light beam).

Group 2 – Application of Nano ZnO gel followed by sham PCT.

Group 3 – Application of placebo gel followed by PCT.

Group 4 – Application of Nano ZnO gel followed by PCT.

The interventions for each group were performed on all periodontal pockets in the assigned quadrant at baseline, at the end of the first week and 1 month after the first session. The interventions were allocated to the quadrants using sealed opaque envelopes. On the day of intervention, each subject chose one envelope to detect their randomized allocation. In each quadrant, the tooth with the deepest PPD was chosen as the test site.

The microbiological sampling and the assessment of clinical parameters were performed at baseline (prior to SRP), at 1 month from baseline and 3 months from it. These procedures were carried out by a single examiner who was blinded to all the study groups. The intra-operator reliability test carried out to establish reproducibility of the results was found to be substantial (Cohen’s kappa-0.8), with an observed percentage agreement of more than 75%.

### Primary outcome measure

Real time Quantitative Polymerase Chain Reaction (RT- qPCR) was carried out to detect the 16S rRNA gene of *Porphyromonas gingivalis* (*P. gingivalis)*. The *P. gingivalis* level was the primary outcome measure assessed.

### Secondary outcome measures

**Figure 3:**
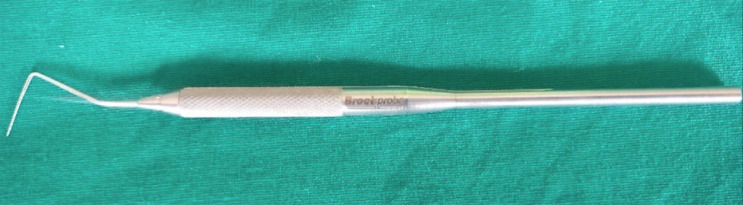
Brockprobe

The secondary outcomes assessed were the Plaque index^8^(PI; Silness and Loe, 1964), Gingival Index^8^(GI; Loe and Silness 1962), Gingival Bleeding Index^9^(GBI, Ainamo & bay 1975), PPD^10^ and CAL^10^, measured at the test sites in all 4 quadrants of each recruited subject using (Brockprobe^TM 10^) U.S Patent # 5,000,683, Brockport Industries, Hackettstown, NJ) (Figure 3).

All the subjects recruited for the study and the operator assessing the treatment outcomes were blinded throughout the study period.

### Microbiological sampling

**Figure 4:**
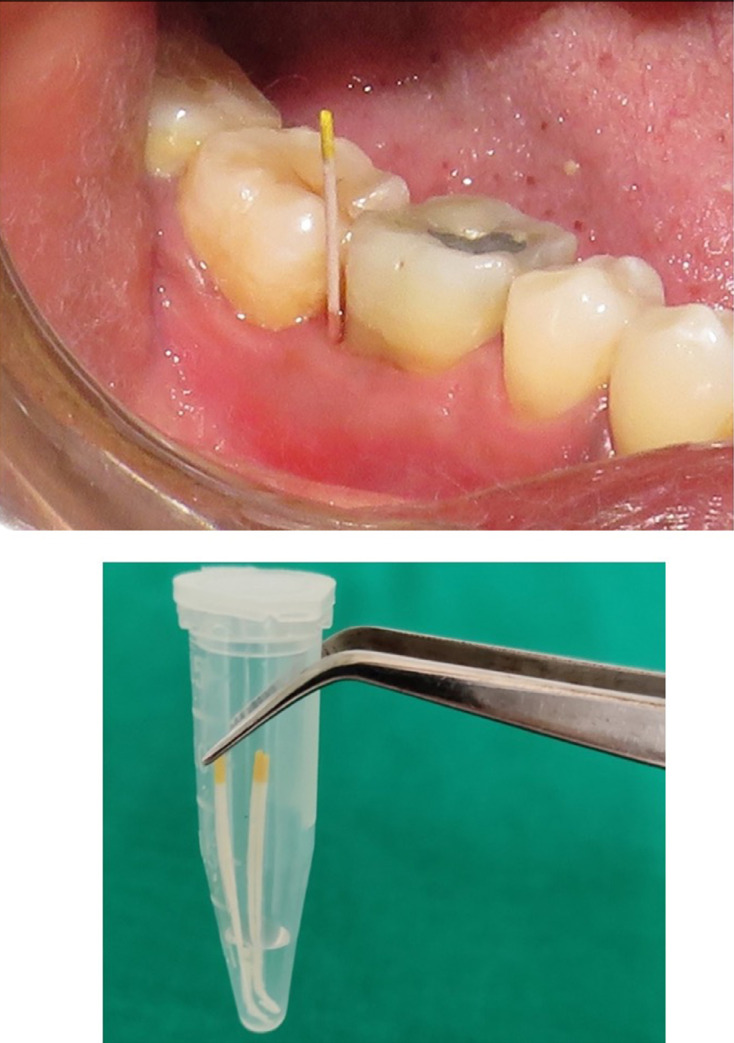
Microbiological sampling

After being careful isolation, the supragingival plaque was removed using a sterile curette. Pooled subgingival plaque samples were collected from the selected site using sterile paper points No. 20 (Figure 4). Each paper point was inserted into the selected site and left there for 20 seconds. The paper points were then transferred to a sterile eppendorf tube containing selective transport media [10x TE (Protenase,DNase,RNase)] (Figure 4) and taken to the laboratory, in order to be evaluated with the real time q-PCR test, which allowed for the estimation of *P. gingivalis* levels. The samples were stored at -70^0^ C until undergoing processing in the laboratory. (New Brunswick Scientific Ultra-Low Temperature Freezer).^11^

### Preparation of test gels

**Figure 5:**
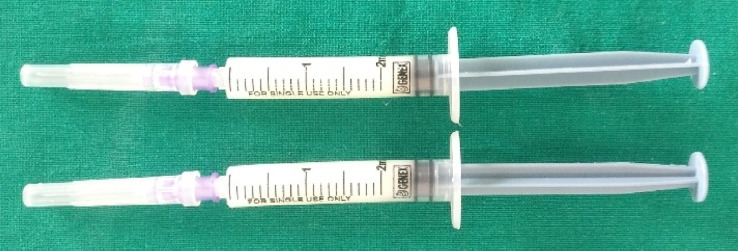
1% Nano ZnO Gel and Placebo Gel

Nano ZnO gel with 1% weight by volume was prepared using bio-hydrothermally synthesized ZnO NPs, which had the aqueous extract of *Emblica Officinalis* (*E. Officinalis*) fruit used as bioreductant. These ZnO NPs were dispersed in a biocompatible gel formulation containing food-grade xanthan gum, agar gelling agent and other pharmaceutical grade excipients (this method is under patenting). The placebo gel contained similar basic ingredients, except for the bio-hydrothermally synthesized ZnO NPs (Figure 5). The biohydrothermally synthesized ZnO NPs were shown to have antimicrobial activity against a few selected oral pathogens in the range of 0.1 mg-0.0125 mg/ml concentration.

### PCT application

The interventions were carried out by a single operator throughout the study period, in order to eliminate inter-operator variability. However, the operator could not be blinded, as this study had a split-mouth design, with all subjects receiving all four interventions.

**Figure 6:**
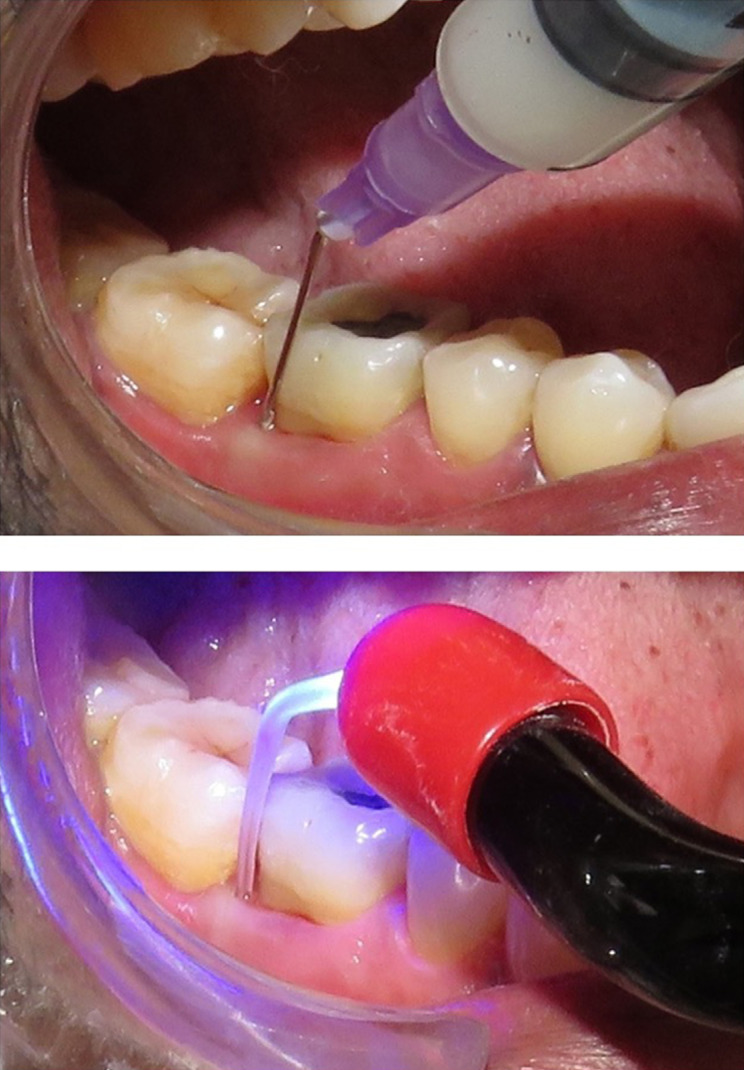
Application of 1 % Nano ZnO gel and light irradiation with curing light

A little less than 1ml of 1% gel formulation (Nano ZnO/ placebo) was applied to the periodontal pocket with a blunt cannula, filling it from its base to its coronal end (Figure 6). The effective amount of ZnO NPs that reached the target site ranged between 0.5-1mg. The gel was kept in the pocket for 5 minutes. A perio tip was attached to the hand piece of the light cure unit (Tulip digital LED curing light, Wavelength: 420-480nm, Light power: 1200mw/cm^2^) and light was activated for 60 seconds, continuously (Figure 6). Following this, the pockets were copiously irrigated with a normal saline solution (0.9% Sodium chloride).

### Microbiological analysis

The DNA Extraction Procedure was carried out using the Modified Proteinase-K method, and the isolated DNA was stored at -20^0^C. Subsequently, the RT-qPCR test was conducted during the microbiological analysis in order to detect the 16S rRNA gene of *P. gingivalis*. The following set of PCR primers, specific to 16SrRNA gene of *P. gingivalis*, were used:^12^

Forward primer: AGG CAG CTT GCC ATA CTG CG

Reverse primer: ACT GTT AGC AAC TAC CGA TGT

The RT-qPCR amplification and detection were performed with the Realplex master cycler (Eppendorf) using a 96-well format. To limit contamination, the reactions were set up in a laminar airflow chamber (Bio-safety cabinet), and run and analyzed in another laboratory, where DNA manipulation was not performed. PCR reactions were carried out in a total volume of 20μl containing 2μl of template DNA, 10μl of Quantitect SYBR green PCR master mix (Qiagen, JJ Biotech, India), and 8pm/μl of each of the *P. gingivalis* specific primers. The conditions for the qPCR reaction were set at 95°C for 3 minutes and 35 cycles of 95°C for 30 seconds, 60°C for 30 seconds and 72°C for 30 seconds. Deionized water was used as negative control. Lastly, fluorescence graphs showing the amplification plot (fluorescence versus cycle numbers) were generated.

### Statistical Analysis

The intragroup comparison for *P. gingivalis* levels and GBI scores was done using Friedman’s test, followed by the Wilcoxon Signed Rank Post hoc Test. The intergroup comparison of *P. gingivalis* and GBI scores was conducted using Wilcoxon Signed rank test. The intragroup comparison of PI, GI, PPD and CAL scores was done using the Repeated Measures of ANOVA and Bonferroni’s Post hoc test. The intergroup comparison of PI, GI, PPD and CAL scores was carried out with Student’s Paired t test.

## Results

All subjects, who had a mean age of 37.9±5.0 years, completed the course of the study. Among all participants, 62.5% were males and 37.5% were females.

### Analysis of microbiological parameters

The statistical intragroup comparison within each of the 4 groups revealed that the mean levels of *P. gingivalis* in Group 1 differed significantly across the timepoints analyzed in the study (P=0.02). In Groups 2, 3 and 4 there were high statistically significant differences (P<0.001) in mean values at all timepoints. While conducting pairwise comparisons between different timepoints in each of the 4 groups, it was found that the mean *P. gingivalis* levels in Group 1 underwent a statistically significant decrease from baseline to month 1 (P=0.04), but significantly rose from month 1 to the end of month 3 (P=0.04). This reduction from baseline to the end of months 3 was, however, not significant. In Group 2, there was a statistically significant decrease in mean values from baseline to month 1 (P=0.01) and from baseline to the end of month 3 (P=0.005), while there was a statistically significant rise from month 1 to the end of month 3 (P=0.001). In Group 3, a statistically significant decrease in mean values occurred from baseline to month 1 (P=0.001) and a highly statistically significant reduction from baseline to the end of month 3 (P<0.001). However, in Group 3, there was a highly statistically significant rise (P<0.001) in mean *P. gingivalis* levels from month 1 to the end of month 3. In Group 4, there was a reduction in mean *P. gingivalis* levels from baseline to month1, from month 1 to the end of month 3 and from baseline to month 3 (P<0.001), all of which were statistically significant (P=0.001) (Table 1).

**Table 1:** Intragroup comparison of mean *P. gingivalis* levels at different time intervals in each study group using Friedman’s Test followed by the Wilcoxon Signed Rank Post hoc Test

Parameter	Groups	Baseline		1 Month		3 Months		P-Value	Wilcoxon Post hoc Test		
		Mean	SD	Mean	SD	Mean	SD		BL vs 1M	BL vs 3M	1M vs 3M
*P. gingivalis*	Group 1	178002.1	216512.4	154843.3	217116.2	177580.3	216788.1	0.02*	0.04*	0.72	0.04*
	Group 2	269618.8	278931.7	232118.8	238037.3	261675	267559.1	<0.001*	0.01*	0.005*	0.001*
	Group 3	160087.5	159362.3	104512.5	104511	124700	123257.9	<0.001*	0.001*	<0.001*	<0.001*
	Group 4	210931.8	249465.7	93279.2	108003.8	82412.5	92443.8	<0.001*	0.001*	<0.001*	0.29

*Statistically significant

The intergroup comparison between the 4 groups showed that their mean *P. gingivalis* levels at baseline and month 1 were comparable, exhibiting no statistically significant differences. The lowest *P. gingivalis* levels at the end of month 3 were those in Group 4, followed by those in Group 3, Group 1 and Group 2, with statistically significant differences between levels in Groups 1 and 4, and between levels in Groups 2 and 4 (P=0.02 and P=0.04 respectively) (Table 2).

**Table 2:** Intergroup comparison of mean *P. gingivalis* levels in different groups at baseline, month 1 and after 3 months, using the Wilcoxon Signed Rank Test

Timepoint	Groups	N	*P. gingivalis*			
			Mean	SD	Mean Diff	P-Value
Baseline	Group 1	16	178002.1	216512.4	-91616.6	0.38
	Group 2	16	269618.8	278931.7		
	Group 1	16	178002.1	216512.4	17914.6	0.8
	Group 3	16	160087.5	159362.3		
	Group 1	16	178002.1	216512.4	-32929.7	0.36
	Group 4	16	210931.8	249465.7		
	Group 2	16	269618.8	278931.7	109531.3	0.3
	Group 3	16	160087.5	159362.3		
	Group 2	16	269618.8	278931.7	58686.9	0.5
	Group 4	16	210931.8	249465.7		
	Group 3	16	160087.5	159362.3	-50844.3	0.72
	Group 4	16	210931.8	249465.7		
1 Month	Group 1	16	154843.3	217116.2	-77275.5	0.35
	Group 2	16	232118.75	238037.28		
	Group 1	16	154843.3	217116.2	50330.8	0.96
	Group 3	16	104512.5	104511		
	Group 1	16	154843.3	217116.2	61564.1	0.13
	Group 4	16	93279.2	108003.8		
	Group 2	16	232118.75	238037.28	127606.3	0.1
	Group 3	16	104512.5	104511		
	Group 2	16	232118.75	238037.28	138839.6	0.06
	Group 4	16	93279.2	108003.8		
	Group 3	16	104512.5	104511	11233.3	0.76
	Group 4	16	93279.2	108003.8		
3 Months	Group 1	16	177580.3	216788.1	-84094.8	0.36
	Group 2	16	261675	267559.1		
	Group 1	16	177580.3	216788.1	52880.3	0.57
	Group 3	16	124700	123257.9		
	Group 1	16	177580.3	216788.1	95167.8	0.02*
	Group 4	16	82412.5	92443.8		
	Group 2	16	261675	267559.1	136975	0.07
	Group 3	16	124700	123257.9		
	Group 2	16	261675	267559.1	179262.5	0.04*
	Group 4	16	82412.5	92443.8		
	Group 3	16	124700	123257.9	42287.5	0.3
	Group 4	16	82412.5	92443.8		

*Statistically significant

### Analysis of clinical parameters

At baseline, the values of all clinical parameters were comparable in all study groups.

While from baseline to the end of month 3 all groups showed decreasing trends in the mean values of all recorded clinical parameters, Group 4 showed decreases in mean PI and GI scores from baseline to month 1, from baseline to the end of month 3—both of which were highly statistically significant—, and from month 1 to the end of month 3. In Groups 1, 2 and 3 there was a rise in mean values from month 1 to the end of month 3—and this rise was statistically significant in Groups 2 and 3 (Table 3). A similar trend was observed in GBI scores from month 1 to the end of month 3 (Table 4). Mean PPD and CAL values rose from month 1 to the end of month 3 in Groups 1, 2, 3 and decreased in Group 4, which makes the difference from baseline to the end of month 3 continue to be significant (Table 3).

**Table 3:** Intragroup comparison of mean PI scores, GI scores, PPD and CAL at different time intervals in each study group using the Repeated Measures of ANOVA Test followed by Bonferroni’s Post hoc Test

Parameter	Group	Baseline		1 Month		3 Months		P-Value	Bonferroni's Post hoc Test		
		Mean	SD	Mean	SD	Mean	SD		BL vs 1M	BL vs 3M	1M vs 3M
PI	Group 1	2.44	0.43	1.27	0.99	2.09	1.04	0.01*	<0.001*	0.85	0.19
	Group 2	2.53	0.43	0.78	0.68	2.23	0.94	<0.001*	<0.001*	0.98	0.006*
	Group 3	2.31	0.46	0.72	0.73	1.81	0.72	<0.001*	<0.001*	0.1	0.003*
	Group 4	2.39	0.5	1.27	0.57	0.61	0.69	<0.001*	<0.001*	<0.001*	0.06
GI	Group 1	2.31	0.46	1.31	0.86	2.11	0.87	0.01*	<0.001*	1	0.14
	Group 2	2.44	0.43	0.78	0.55	2.16	1.06	<0.001*	<0.001*	1	0.006*
	Group 3	2.39	0.5	0.73	0.45	1.61	0.82	<0.001*	<0.001*	0.009*	0.02*
	Group 4	2.53	0.43	1.27	0.82	0.69	0.67	<0.001*	<0.001*	<0.001*	0.28
PPD	Group 1	4.59	1.24	4.07	1.32	4.56	1.22	0.02*	0.002*	1	0.15
	Group 2	4.35	1.08	3.66	1.02	4.23	0.93	0.01*	<0.001*	1	0.1
	Group 3	4.56	1.27	3.67	0.91	3.96	0.98	0.007*	<0.001*	0.18	0.44
	Group 4	4.44	1.05	3.68	0.88	3.16	0.94	<0.001*	<0.001*	<0.001*	0.13
CAL	Group 1	5.28	1.63	3.47	1.72	4.63	1.32	0.004*	<0.001*	0.62	0.14
	Group 2	4.99	1.02	2.84	0.87	4.63	1.54	<0.001*	<0.001*	1	0.009*
	Group 3	5.06	1.16	2.93	0.84	4.37	1.39	<0.001*	<0.001*	0.04*	0.002*
	Group 4	5.38	1.48	3.53	0.53	3.13	0.78	<0.001*	<0.001*	<0.001*	0.43

*Statistically significant

**Table 4:** Intragroup comparison of mean GBI scores at different time intervals in each study group using Friedman’s Test followed by the Wilcoxon Signed Rank Post hoc Test

Parameter	Groups	Baseline		1 Month		3 Months		P-Value	Wilcoxon Post hoc Test		
		Mean	SD	Mean	SD	Mean	SD		BL vs 1M	BL vs 3M	1M vs 3M
GBI	Group 1	83.34	14.89	59.38	21.93	78.12	23.34	0.002*	<0.001*	1	0.06
	Group 2	82.29	14.22	48.23	24.76	82.17	20.28	0.001*	<0.001*	1	0.008*
	Group 3	84.38	14.22	36.45	24.5	63.54	28.69	<0.001*	<0.001*	0.03*	0.01*
	Group 4	85.41	13.43	48.96	21.49	36.46	28.68	<0.001*	<0.001*	<0.001*	0.47

*Statistically significant

During the intergroup comparison at the end of month 3, it was found that Group 4 had the lowest mean PI and GI values, followed by Group 3, Group 2 and Group 1, with a statistically significant difference occurring between Groups 3 and 4, and a highly statistically significant difference occurring between Groups 1 and 4, and between Groups 2 and 4 (Table 5). At the end of month 3, Group 4 had the lowest mean GBI value, which differed significantly from the values in the other groups (Table 6). A similar trend was observed for CAL values. In contrast, although PPD values in Group 4 significantly differed from those presented by other groups, it was Group 2 that had the lowest mean PPD values at the end of month 3. (Table 5)

**Table 5:** Intergroup comparison of mean PI scores, GI scores, PPD and CAL in different groups at baseline, month 1 and after 3 months, using Student’s Paired t Test

Timepoint	Groups	N	PI				GI				PPD				CAL			
			**Mean**	**SD**	**Mean Diff**	**P-Value**	**Mean**	**SD**	**Mean Diff**	**P-Value**	**Mean**	**SD**	**Mean Diff**	**P-Value**	**Mean**	**SD**	**Mean Diff**	**P-Value**
**Baseline**	Group 1	16	2.44	0.43	-0.09	0.58	2.31	0.46	-0.13	0.43	4.59	1.24	0.24	0.38	5.28	1.63	0.29	0.44
	Group 2	16	2.53	0.43			2.44	0.43			4.35	1.08			4.99	1.02		
	Group 1	16	2.44	0.43	0.13	0.43	2.31	0.46	-0.08	0.71	4.59	1.24	0.03	0.94	5.28	1.63	0.23	0.67
	Group 3	16	2.31	0.46			2.39	0.5			4.56	1.27			5.06	1.16		
	Group 1	16	2.44	0.43	0.05	0.78	2.31	0.46	-0.22	0.24	4.59	1.24	0.15	0.75	5.28	1.63	-0.09	0.87
	Group 4	16	2.39	0.5			2.53	0.43			4.44	1.05			5.38	1.48		
	Group 2	16	2.53	0.43	0.22	0.24	2.44	0.43	0.05	0.78	4.35	1.08	-0.21	0.41	4.99	1.02	-0.06	0.83
	Group 3	16	2.31	0.46			2.39	0.5			4.56	1.27			5.06	1.16		
	Group 2	16	2.53	0.43	0.14	0.29	2.44	0.43	-0.09	0.58	4.35	1.08	-0.09	0.76	4.99	1.02	-0.38	0.42
	Group 4	16	2.39	0.5			2.53	0.43			4.44	1.05			5.38	1.48		
	Group 3	16	2.31	0.46	-0.08	0.71	2.39	0.5	-0.14	0.29	4.56	1.27	0.12	0.62	5.06	1.16	-0.32	0.36
	Group 4	16	2.39	0.5			2.53	0.43			4.44	1.05			5.38	1.48		
1 Month	Group 1	16	1.27	0.99	0.49	0.04*	1.31	0.86	0.53	0.03*	4.07	1.32	0.41	0.09	3.47	1.72	0.63	0.19
	Group 2	16	0.78	0.68			0.78	0.55			3.66	1.02			2.84	0.87		
	Group 1	16	1.27	0.99	0.55	0.11	1.31	0.86	0.58	0.03*	4.07	1.32	0.4	0.3	3.47	1.72	0.54	0.2
	Group 3	16	0.72	0.73			0.73	0.45			3.67	0.91			2.93	0.84		
	Group 1	16	1.27	0.99	0	1	1.31	0.86	0.05	0.88	4.07	1.32	0.39	0.36	3.47	1.72	-0.06	0.9
	Group 4	16	1.27	0.57			1.27	0.82			3.68	0.88			3.53	0.53		
	Group 2	16	0.78	0.68	0.06	0.77	0.78	0.55	0.05	0.73	3.66	1.02	-0.01	0.98	2.84	0.87	-0.09	0.59
	Group 3	16	0.72	0.73			0.73	0.45			3.67	0.91			2.93	0.84		
	Group 2	16	0.78	0.68	-0.49	0.06	0.78	0.55	-0.49	0.11	3.66	1.02	-0.01	0.97	2.84	0.87	-0.69	0.01*
	Group 4	16	1.27	0.57			1.27	0.82			3.68	0.88			3.53	0.53		
	Group 3	16	0.72	0.73	-0.55	0.04*	2.16	1.06	1.47	<0.001*	3.67	0.91	-0.01	0.98	2.93	0.84	-0.6	0.04*
	Group 4	16	1.27	0.57			0.69	0.67			3.68	0.88			3.53	0.53		
3 Months	Group 1	16	2.09	1.04	-0.14	0.62	2.11	0.87	-0.05	0.9	4.56	1.22	0.33	0.39	4.63	1.32	0	1
	Group 2	16	2.23	0.94			2.16	1.06			4.23	0.93			4.63	1.54		
	Group 1	16	2.09	1.04	0.28	0.47	2.11	0.87	0.5	0.09	4.56	1.22	0.6	0.09	4.63	1.32	0.26	0.51
	Group 3	16	1.81	0.72			1.61	0.82			3.96	0.98			4.37	1.39		
	Group 1	16	2.09	1.04	1.49	<0.001*	2.11	0.87	1.42	<0.001*	4.56	1.22	1.39	0.002*	4.63	1.32	1.5	0.001*
	Group 4	16	0.61	0.69			0.69	0.67			3.16	0.94			3.13	0.78		
	Group 2	16	2.23	0.94	0.42	0.21	2.16	1.06	0.55	0.08	4.23	0.93	0.27	0.33	4.63	1.54	0.26	0.6
	Group 3	16	1.81	0.72			1.61	0.82			3.96	0.98			4.37	1.39		
	Group 2	16	2.23	0.94	1.63	<0.001*	2.16	1.06	1.47	<0.001*	4.23	0.93	1.06	0.004*	4.63	1.54	1.5	0.007*
	Group 4	16	0.61	0.69			0.69	0.67			3.16	0.94			3.13	0.78		
	Group 3	16	1.81	0.72	1.2	0.001*	1.61	0.82	0.92	0.002*	3.96	0.98	0.79	0.03*	4.37	1.39	1.24	0.004*
	Group 4	16	0.61	0.69			0.69	0.67			3.16	0.94			3.13	0.78		

*Statistically significant

**Table 6:** Intergroup comparison of mean GBI scores in different groups at baseline, month 1 and after 3 months, using the Wilcoxon Signed Rank Test

Timepoint	Groups	N	GBI			
			**Mean**	**SD**	**Mean Diff**	**P-Value**
Baseline	Group 1	16	83.34	14.89	1.04	0.88
	Group 2	16	82.29	14.22		
	Group 1	16	83.34	14.89	-1.04	0.84
	Group 3	16	84.38	14.22		
	Group 1	16	83.34	14.89	-2.08	0.67
	Group 4	16	85.41	13.43		
	Group 2	16	82.29	14.22	-2.08	0.73
	Group 3	16	84.38	14.22		
	Group 2	16	82.29	14.22	-3.12	0.51
	Group 4	16	85.41	13.43		
	Group 3	16	84.38	14.22	-1.04	0.79
	Group 4	16	85.41	13.43		
1 Month	Group 1	16	59.38	21.93	11.14	0.19
	Group 2	16	48.23	24.76		
	Group 1	16	59.38	21.93	22.93	0.01*
	Group 3	16	36.45	24.5		
	Group 1	16	59.38	21.93	10.42	0.25
	Group 4	16	48.96	21.49		
	Group 2	16	48.23	24.76	11.78	0.19
	Group 3	16	36.45	24.5		
	Group 2	16	48.23	24.76	-0.73	0.94
	Group 4	16	48.96	21.49		
	Group 3	16	36.45	24.5	-12.51	0.19
	Group 4	16	48.96	21.49		
3 Months	Group 1	16	78.12	23.34	-4.05	0.54
	Group 2	16	82.17	20.28		
	Group 1	16	78.12	23.34	14.58	0.12
	Group 3	16	63.54	28.69		
	Group 1	16	78.12	23.34	41.66	<0.001*
	Group 4	16	36.46	28.68		
	Group 2	16	82.17	20.28	18.63	0.08
	Group 3	16	63.54	28.69		
	Group 2	16	82.17	20.28	45.71	<0.001*
	Group 4	16	36.46	28.68		
	Group 3	16	63.54	28.69	27.09	0.01*
	Group 4	16	36.46	28.68		

*Statistically significant

## Discussion

The desirable effects of locally administered anti-infective agents can be increased with the application of new treatment modalities, including PDT. However, several agents that are currently used in treatments tend to produce harmful effects. Thus, the present *in vivo*, randomized, controlled clinical study evaluated the efficacy of PCT using bio-hydrothermally synthesized Nano ZnO gel in combination with visible light as an adjunct to SRP in the treatment of periodontitis.

Despite being applied repeatedly, the test gel containing ZnO NPs was well tolerated by all patients in the study. This may be attributed to the fact that the gel was synthesized with a “bio-synthetic approach” so as to minimize any potential risks or hazards.

A previous study by the authors evaluated the potential toxicity of ZnO NPs synthesized using *E. officinalis* aqueous extract in combating Red Blood Cells (RBCs) isolated from chick blood, balb 3T3 mice fibroblast cell lines and *Bombyx mori* silkworm (*in vivo*). For this, ZnO powder used in Clinical Dentistry was used as a control. The results showed that ZnO NPs synthesized using *E. officinalis* aqueous extract had no toxicity against RBCs and, even at the highest tested concentration, only inhibited the growth of less than 35% of mice fibroblast cell lines. The substance used in the control experiment, a ZnO powder that is routinely used in Dentistry, showed moderate toxicity against RBCs and fibroblast cell lines. Noticeably, the ZnO NPs caused a mortality rate of < 27% in *B mori* silkworm larvae, whereas the ZnO powder (control) caused a mortality rate of ~97% in silkworm larvae. Thus, *E. officinalis* used in formulating Nano ZnO gel causes less ecotoxic effects. The ZnO powder that has been long employed in dental therapeutics needs to be further investigated, since it is synthesized using chemical reduction methods and can generate ecotoxic sequels when discarded into the environment.^13^

*P. gingivalis* is often referred to as the keystone pathogen in the etiopathogenesis of periodontitis in humans. It is resistant to subgingival debridement due to its ability to invade pocket epithelium and connective tissue. In a study conducted by Talebi, et al.^11^ (2016), positive results were observed for SRP alone after one month, but within three months, these outcomes reverted and got worse.^5^ This may be attributed to the insufficient instrumentation of inaccessible areas and to the recolonization of the subgingival areas from other oral ecological niches.^1^In our study, both at month 1 and after 3 months, all adjunctive therapies seemed to generate greater improvement than conventional SRP alone—and PCT, specifically, caused a reduction in subgingival levels of *P. gingivalis* that, at both time points, seemed to be much larger than the ones caused by its individual components, Nano ZnO, visible light and SRP alone. The results of our research were similar to those obtained in previous studies in which test groups receiving adjunctive PDT underwent a greater reduction in subgingival *P. gingivalis* levels.^14-16^A systematic review by Akram, et al.^17^ (2016) concluded that adjunctive PDT may provide synergistic effects with SRP in improving therapeutic outcomes. However, its antibacterial efficacy remains a subject of debate. In our study, Groups 3 and 4 showed no statistically significant differences at the end of month 3, but considering the fact that Group 3 presented a statistically significant rise in *P. gingivalis* levels from month 1 to the end of month 3, PCT is suggested to produce better sustained effects on the subgingival plaque.

All clinical parameters were assessed with the aid of Brockprobe^TM^, a 2^nd^ generation pressure sensitive probe with William’s markings (1, 2, 3, 5, 7, 8, 9, 10mm), which allowed results to be reproducible at any point in time.^10^

The mean PI scores obtained in our study indicate that all subjects maintained comparable oral hygiene levels throughout the study period, while the sustained effects presented by Group 4 may have resulted from the beneficial antimicrobial effects of PCT using Nano ZnO gel and visible light. The mean GI values indicate that a prolonged beneficial effect was provided by PCT using Nano ZnO and visible light, which caused sustained effects that likely contributed to reducing plaque and subsequently decreasing gingival inflammation, resulting in some amount of tissue shrinkage and PPD reduction as well. Results similar to ours were observed by Lulic, et al.^18^ (2009), Ge, et al.^19^ (2011), Müller Campanile^20^ (2015), Franco, et al.^21^ (2014), and Monzavi, et al.^22^ (2016), who reported a statistically significant reduction in bleeding on probing after the repeated application of adjunctive PDT. The reduction in mean GBI scores can be associated with the improvement in GI, which further supports that PCT using ZnO and visible light generates beneficial effects.

At both follow-up intervals, all adjunctive therapies seemed to produce greater PPD reduction and CAL improvement than conventional SRP alone, but after 3 months, the changes caused by PCT seemed to be greater than those caused by its individual components, Nano ZnO and visible light and SRP alone. This reduction in mean PPD can be associated with improvements in gingival status and in the periodontal attachment, which also supports that PCT has beneficial effects. A systematic review and meta-analysis conducted by Azaripour, et al.^23^ (2018) concluded that using PDT as an adjunct to SRP results in significant PPD reduction, which becomes evident at the end of months 3 and 6 of the treatment of chronic periodontitis. However, several studies have found that a single application of PDT caused no significant beneficial effects on PPD after 3 months.^24-26^In contrast, a few other studies showed that the repeated application of PDT was more effective in reducing PPD than its single application, both at month 1 and after 3 months of treatment, which was in accordance with our results.^18,20,27^In studies conducted by Müller Campanile^20^ (2015) and Sreedhar, et al.^27^ (2015), the repeated application of PDT resulted in a statistically significant gain in CAL as compared to SRP alone at various time intervals—and these results are also in accordance with those of our study. This increase in the mean CAL can be attributed to the positive effect of PCT on periodontal attachment.

Considering all clinical and microbiological parameters assessed in this study, we found that all four treatment modalities produced significant improvement after 1 month. Similar results were obtained with SRP alone in several clinicomicrobiological studies, as discussed above. We observed that repeated application of Nano gel containing ZnO NPs as an adjunct to SRP resulted in better periodontal variables at month 1 than the use of SRP alone. At 3 months, both treatment modalities produced comparable results. The immediate results showcased by Nano ZnO gel may be attributed to its inherent antibacterial properties, which have been exploited by several other applications, including that of periodontal pack and intracanal medicaments.^28^

At the end of month 1, it was found that the use of visible light as an adjunct to SRP resulted in better treatment outcomes than the employment of SRP alone or with adjunctive Nano ZnO gel. The various *in vitro* studies conducted by Feuerstein, et al.^29^ (2004), Kotoku, et al.^30^ (2009), Kim, et al.^31^ (2013) and Song, et al.^32^ (2013) evaluated the antimicrobial effect of visible light in the blue range on *P. gingivalis* and observed that bacterial activity was inhibited by 80 - 100%.^29-32^A systematic review carried out by Pummer, et al.^33^ (2017) evaluated the *in vitro* antimicrobial activity of visible light and concluded that *P. gingivalis* was susceptible to blue and red light irradiation, although effects were greater with the former, which had a longer wavelength and could penetrate tissues to a deeper extent. However, at 3 months, the outcomes of our study did not show a statistically significant intergroup difference.

Both Nano ZnO and visible light caused a reversal phenomenon comparable to that of SRP in all parameters from months 1-3, despite generating significant effects until month 1. This may be attributed to the repeated application of the specific adjunctive treatment modality, which enforced immediate effects that could not be sustained until the end of month 3.

It was observed that although PCT had a slow initial effect (which was assessed the end of month 1), it was the only treatment modality that caused a sustained progressive improvement in all the outcome measures tested between month 1 and the end of month 3—it caused the periodontal status to significantly improve from baseline to the end of month 3.

### Limitations

Despite trying to maintain a quality study protocol, incorporating measures such as randomization, the blinding of subjects and outcome assessors, as well as using standardized probing force to assess clinical parameters and high quality RT-qPCR to quantify *P. gingivalis*, our study included a small sample size and had a short-term follow-up. The study population was not subcategorized to evaluate the differential effects in moderate and deep pockets, and other periodontopathogens chiefly associated with periodontitis were also not considered.

## Conclusion

All four treatment modalities employed in the study, aided by the meticulous oral hygiene maintenance of the participants, resulted in an overall improvement of all clinical parameters and microbiological profile assessed after 3 months. At the end of month 1, it was found that the repeated application of PCT as an adjunct to SRP, using bio-hydrothermally synthesized Nano ZnO gel in combination with visible light in the range of 420-480 nm, produced a significantly better short-term improvement in treatment outcomes than SRP alone—but this difference between the two treatments was not sustained until the end of month 3. All groups except for the one that received the adjunctive PCT application experienced an increase in *P. gingivalis* levels at the end of month 3 (compared to month 1). The sustained improvement in all clinical parameters and the reduction in *P. gingivalis* levels from month 1 to the end of month 3 after the adjunctive application of PCT may be attributed to the synergistic effect of both Nano ZnO gel and visible light, which may have eliminated the etiology from all inaccessible micro-environments.

Longitudinal studies with a larger sample size and a longer follow-up period can further validate the beneficial effects of the adjunctive application of PCT using bio-hydrothermally synthesized Nano ZnO gel and visible light in the range of 420-480 nm in the treatment of periodontitis.
